# Position and sequence conservation in Amniota of polymorphic enhancer HS1.2 within the palindrome of IgH 3'Regulatory Region

**DOI:** 10.1186/1471-2148-11-71

**Published:** 2011-03-15

**Authors:** Pietro D'Addabbo, Moira Scascitelli, Vincenzo Giambra, Mariano Rocchi, Domenico Frezza

**Affiliations:** 1 Department of Genetics and Microbiology, University of Bari, Bari, Italy; 2 Department of Botany, University of British Columbia, Vancouver, British Columbia, Canada; 3 Terry Fox Laboratory, BC Cancer Agency, Vancouver, British Columbia, Canada; 4 Department of Biology, University of Roma Tor Vergata, Viale della ricerca scientifica. 00133 Roma, Italy

## Abstract

**Background:**

The Immunoglobulin heavy chain (IgH) 3' Regulatory Region (3'RR), located at the 3' of the constant alpha gene, plays a crucial role in immunoglobulin production. In humans, there are 2 copies of the 3'RR, each composed of 4 main elements: 3 enhancers and a 20 bp tandem repeat. The single mouse 3'RR differs from the two human ones for the presence of 4 more regulative elements with the double copy of one enhancer at the border of a palindromic region.

**Results:**

We compared the 3'RR organization in genomes of vertebrates to depict the evolutionary history of the region and highlight its shared features. We found that in the 8 species in which the whole region was included in a fully assembled contig (mouse, rat, dog, rabbit, panda, orangutan, chimpanzee, and human), the shared elements showed synteny and a highly conserved sequence, thus suggesting a strong evolutionary constraint. In these species, the wide 3'RR (~30 kb in human) bears a large palindromic sequence, consisting in two ~3 kb complementary branches spaced by a ~3 kb sequence always including the HS1.2 enhancer. In mouse and rat, HS3 is involved by the palindrome so that one copy of the enhancer is present on each side. A second relevant feature of our present work concerns human polymorphism of the HS1.2 enhancer, associated to immune diseases in our species. We detected a similar polymorphism in all the studied Catarrhini (a primate parvorder). The polymorphism consists of multiple copies of a 40 bp element up to 12 in chimpanzees, 8 in baboons, 6 in macaque, 5 in gibbons, 4 in humans and orangutan, separated by stretches of Cytosine. We show specific binding of this element to nuclear factors.

**Conclusions:**

The nucleotide sequence of the palindrome is not conserved among evolutionary distant species, suggesting pressures for the maintenance of two self-matching regions driving a three-dimensional structure despite of the inter-specific divergence at sequence level. The information about the conservation of the palindromic structure and the settling in primates of the polymorphic feature of HS1.2 show the relevance of these structures in the control and modulation of the Ig production through the formation of possible three-dimensional structures.

## Background

**Figure 1:**
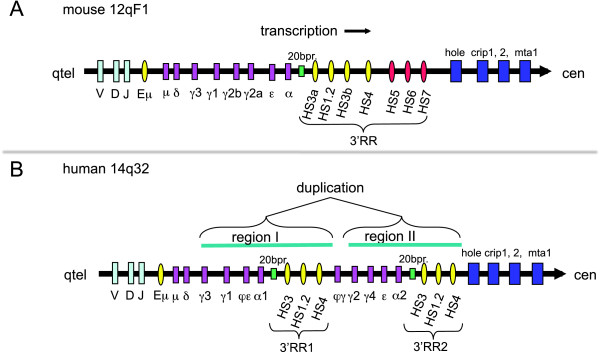
**Schematic map of the mouse and human IgH genetic cluster**. The IgH cluster and the closest genes (dark blue) are highly conserved in the mouse and human chromosomes. The same transcription direction was also detected in both species, with a "telomere towards centromere" transcription versus. The mouse IgH cluster harbors 8 constant genes and one copy of the 3' Regulatory Region (3'RR) with 7 enhancers, 3 of them (HS5, HS6, HS7) with possible insulator function at the 3' boundary of the cluster. The human IgH region has some differences for the constant genes since a wide duplication of gamma, epsilon and alpha genes occurred in the ancestor of Hominoidea. This duplication included also the 3'RR at the 3' of the constant alpha gene. Both the two copies of the 3'RR in humans have just three enhancers. In fact, the human loci do not harbor both the duplicated HS3.B enhancer and the insulators present in the mouse locus.

The immunoglobulin genes appeared, during evolution, in vertebrates. Because of their increasing physiological relevance, the evolution of these genes in fish, amphibian, birds and mammals witnessed several series of duplications that ended in adding copies and complexity to these genes. The class switch, in particular, was of importance in producing diversity[1], and constituted a crucial step in B cell maturation[2]. The region involved by the somatic rearrangements, allowing the class switch, is the Immunoglobulin heavy chain (IgH) locus. This domain is in a single copy in the genome of most extant species (as example, see the mouse locus in Figure 1A). Hominoidea (human, chimpanzee, gorilla and gibbon) are an exception, because the constant genes of the IgH locus underwent duplication in their common ancestor[3]. Studies in humans have shown that the duplication of the IgH locus included the Regulatory Region (3'RR) located immediately downstream of the constant alpha exons[2]. Portions of the 3'RR were first cloned in 1990 and 1991[4,5] but only later were fully assembled as a complete contig sequence because they are repeats-rich unstable regions, moreover containing palindromic sequences[6,7]. In humans, the 2 copies of this 3' Regulatory Region (3'RR) have been reported as 3'RR1 and 3'RR2 (Figure 1B)[8]. Each human 3'RR copy harbors three different enhancers. The mouse and rat 3'RR possess 4 more boundary regulatory regions instead. Their existence in other organisms may be hypothesized but have not been demonstrated yet[2] (Figure 1A).

**Figure 2:**
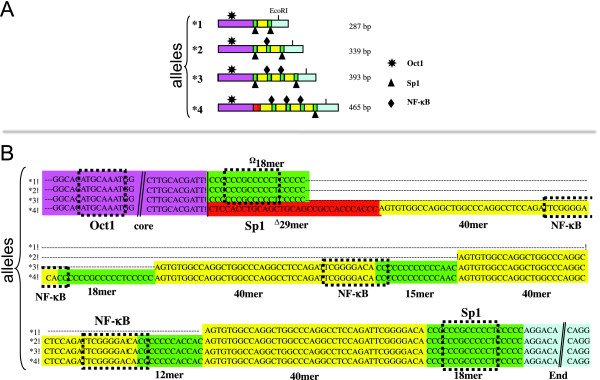
**The polymorphisms of the human enhancer HS1.2**. (A) The schematic representation of the human alleles shows the different invariant and variable elements. Up to now 6 different alleles are reported in GenBank, each one identified for the number of copies (*1, *2, *3, *4) of the 40 bp element (40 mer, in yellow). More variability was added by the presence of either a short stretch of cytosines (18 bp, in green) or a different larger element (29 bp, in red) between the enhancer core (in fuchsia) and the first 40 mer. The 3' end of the enhancer (in light blue) also is highly conserved. The green stretches of cytosines parting the 40 mer ranged between 12 and 18 bp. (B) The sequence alignment of four alleles of HS1.2 enhancer sequences colored according to the schematic elements reported in the upper part. The binding for the Oct1, Sp1 and NF-κB factors are reported on the sequence as determined by EMSA experiments.

The 3'RR has a crucial role in recruiting transcription factors for the initiation of germ line transcription of the constant genes to induce IgH switch[9]. The role of the 3'RR enhancers was studied with mice transgenic for the c-myc translocation, showing the active role of HS3.B and HS4 in peripheral B-cell lymphomas progression and not in pro-B lymphomas[10,11]. Relevant studies demonstrate by chromosome conformation capture techniques the presence of a three-dimensional structure originated by a loop among Regulatory Regions during class switch recombination[12]. New studies on 3'RR transgenic deleted mice report impairment of class switch and Ig expression [13,14]. Activation of the mouse 3'RR begins with selective demethylation of enhancers[15]. Binding sites variation affecting the enhancers sequence can lead to different epigenetic changes and bring cells to differently act. In our recently performed population studies we found, in fact, that some of the 3'RR enhancer HS1.2 variants (Figure 2) were associated to a higher risk for autoimmune diseases onset and other immune-disorders as IgA defect, systemic sclerodermia, Rheumatic arthritis, Psoriasis and Celiac disease [16-20]. We hypothesized that the cause was a change of a binding consensus for NF-κB and other transcription factors as "*in silico*" predicted or experimentally determined [16,21]. In humans the presence of an allele with the NF-κB consensus site was associated to increased haematic concentration of IgM, suggesting a contribution to the mechanism of class switch[16,17].

Little is known on the presence and organization of the 3'RR in Amniota other than human and mouse[22,23]. Comparative studies of this regulatory region can provide hypotheses on which elements are crucial with respect to their function[24]. To fill the gap, we investigated the genomic organization of this region taking advantage of the sequence data present in GenBank and Trace Archive (see Methods below). The most relevant achievement of the analysis was the discovering that the palindrome surrounding the HS1.2 enhancer is present in every mammal species for which enough sequence data were available. This finding has important implication on the understanding of HS1.2 functioning. In addition, our data supports the view that HS1.2 polymorphisms are widely spread in the primate parvorder of Catarrhini (Cercopithecoidea and Hominoidea). The improvement on the comparative studies on the non coding genome is a relevant task for new insight in the epigenetic and mechanisms of genome regulation[25].

## Results

### 3'RR genomic organization

The mouse 3'RR region contains 7 enhancers (HS3.A-HS1.2-HS3.B-HS4-HS5-HS6-HS7), while human has only three enhancers (Figure 1). For this reason the mouse was used as the reference genome for the preliminary analysis. The IgA class exons are the transcripted sequence closest to the 3'RR, so we included a portion of this DNA in our analysis. It is to keep in mind that the IgA was the last class to appear during the evolution of IgH, because it is just shared among Amniota species (reptiles, bird and mammals)[26]. Finally, we surveyed also a satellite repeat. This is a conserved stretch of DNA (812 bp in human) composed of tandemly repeated 20 bp element and located inside the 3'UTR of the IgA gene, close to the 3'RR enhancer.

The "Comparative Genomics" tracks of the UCSC mouse genome browser http://genome.ucsc.edu/cgi-bin/hgGateway?org=Mouse report graphical representations of Lastz comparison http://www.bx.psu.edu/miller_lab/ between mouse and each one of 19 Amniota genomes (rat, guinea pig, rabbit, human, chimp, orangutan, rhesus, marmoset, panda, dog, cat, horse, elephant, cow, pig, opossum, platypus, lizard, chicken). Some of these assemblies, human and mouse in particular, are very accurate. At the contrary some others are based on relatively low sequence coverage, with several unresolved gaps. This peculiar consideration has to be kept in mind when dealing with negative results of sequences comparison among genomes drafts.

**Figure 3:**
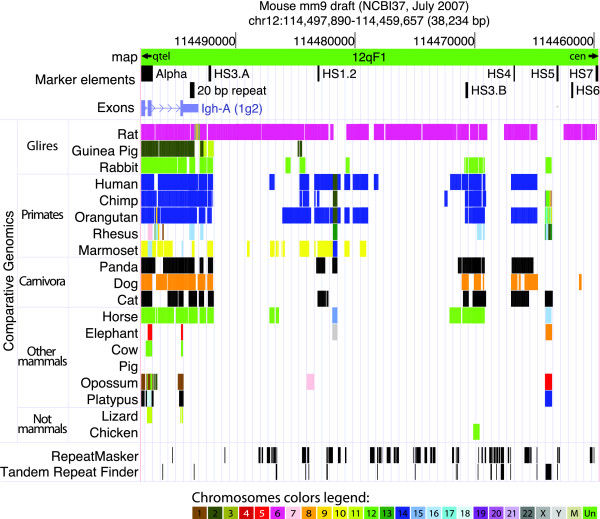
**Contig sequence alignment of the 3'RR of vertebrate species with completed genome sequence**. This figure shows the region chr12:114,459,657-114,497,890 of the UCSC Mouse Genome Browser (mm9 draft). The comparative genomic tracks were inspected and the graphic was edited as in this figure. The species common names are listed in the first column on the left. The black blocks in the "Mouse main elements" section localize the positions of the chosen tags, mapped by the BLAT tool of the site. The colored blocks in the "Comparative Genomics" section show the most similar region in each genome of the species listed, versus the mouse genome. The color of each block represents a portion of a chromosome (see the chromosome color legend below the graphic) of the species similar to that portion of the mouse chromosome 12. The "RepeatMasker" and "Tandem Repeat Finder" sections show the position of repeats identified by the two homonymous algorithms in this portion of mouse genome.

Results of our search for the mouse 3'RR main elements in Amniota genomes are summarized in Figure 3. The full set of elements present in mouse (IgH alpha exons, 20 bp tandem repeat and 7 enhancers) was detected only in the rat genome. The HS5, HS6, and HS7 were always absent in all the remaining species. The HS3, HS1.2, and HS4 set was detected with certainty in mouse, rat, dog, rabbit, panda, human, chimpanzee, and orangutan. HS3 and the 20 bp repeat, were present in 12 mammals, but were undetected in cow, elephant, opossum, and platypus. The region delimited by the alpha exons and HS3.A enhancer and encompassing the 20 bp repeat, appeared to be highly conserved in placental mammals. We remark that, at the contrary, the Alpha marker remains entirely undetected in chicken and pig, because the similarity versus rodent IgA is low even at the level of peptide sequence.

As expected, the search in the Neanderthal genome[27] by inspection of related track in the UCSC human genome browser demonstrated that HS3, HS1.2 and HS4 were also present in the genome of our extinct relative (data not shown).

### Enhancers in the sequenced species

Figure 3 shows comparisons among assembled genome drafts. If the sequence of a specific region is still missing in the genome draft of a particular species, then the comparison versus the mouse genome will not find any match, even though it is expected. These missing sequences, however, can be present in the shot-gun sequences databases, as unassembled short sequences. We searched in GenBank genome-related databases, by BLAST, for the presence of murine (data not shown) and human IgH alpha exons (Table 1). Querying for this transcripted sequence, we tested the method limits. All the 35 positive species were mammals (8 primates). Then we BLASTed the murine (data not shown) and human (Table 1) enhancers against the same positive databases. The analysis showed that at least one 3'RR enhancer was present in 23 species, while all the three enhancers were present in only 8 species (in bold in Table 1), apart from human. The negative findings may be ascribed either to the non-completeness of the available genomic drafts/wgs/htgs databases or to an actual sequences divergence. It is worth noting that the longer map distance between the 3'RR features and the IgA gene, the less species detected (Table 1). Finally, the analysis confirmed that the mouse HS5, HS6, and HS7 were detectable by sequence similarity only in rat (data not shown).

**Table 1:** List of species, other than human, identified by BLAST in wgs and htgs databases

	Alpha		repeat		HS3		HS1,2		HS4	
	**MI**	**QC**	**MI**	**QC**	**MI**	**QC**	**MI**	**QC**	**MI**	**QC**
**Gorilla gorilla - Gorilla (P)**	**94%**	**100%**	**93%**	**100%**	**97%**	**100%**	**94%**	**78%**	**98%**	**100%**
**Pongo abelii - Orangutan (P)**	**94%**	**62%**	**88%**	**99%**	**92%**	**100%**	**92%**	**100%**	**95%**	**99%**
Pan troglodytes - Chimpanzee (P)	93%	87%	87%	100%	99%	100%	95%	100%	-	-
Macaca mulatta - Macaque (P)	89%	100%	82%	100%	93%	100%	90%	80%	-	-
**Felis catus - Cat**	**88%**	**75%**	**76%**	**100%**	**77%**	**66%**	**76%**	**46%**	**69%**	**100%**
Callithrix jacchus - Marmoset (P)	85%	99%	67%	99%	85%	100%	89%	78%	-	-
Sorex araneus - European shrew	81%	71%	70%	52%	-	-	76%	26%	-	-
Otolemur garnettii - Galago (P)	79%	36%	74%	99%	-	-	-	-	-	-
Tarsius syrichta - Tarsier (P)	76%	43%	64%	92%	82%	88%	-	-	-	-
Echinops telfairi - Madagascar hedgehog	75%	42%	-	-	67%	77%	81%	30%	-	-
Choloepus hoffmanni - Sloth	74%	30%	-	-	-	-	-	-	-	-
Tursiops truncatus - Dolphin	73%	98%	76%	99%	77%	84%	73%	45%	-	-
**Canis familiaris - Dog**	**72%**	**99%**	**75%**	**99%**	**74%**	**90%**	**76%**	**47%**	**79%**	**44%**
Microcebus murinus - Lemur (P)	72%	57%	67%	98%	81%	84%	-	-	-	-
Ovis aries - Sheep	72%	43%	-	-	76%	75%	77%	36%	-	-
**Ailuropoda melanoleuca - Panda**	**71%**	**99%**	**79%**	**99%**	**75%**	**82%**	**75%**	**86%**	**70%**	**83%**
Pteropus vampyrus - Large flying fox	71%	99%	67%	61%	78%	84%	-	-	66%	88%
Bos taurus - Cattle	70%	79%	82%	99%	77%	84%	-	-	-	-
Ornithorhynchus anatinus - Platypus	70%	31%	66%	58%	-	-	-	-	-	-
Myotis lucifugus - Little brown bat	70%	21%	69%	86%	-	-	69%	43%	-	-
Equus caballus - Horse	69%	78%	78%	99%	80%	77%	-	-	-	-
Spermophilus tridecemlineatus - Squirrel	69%	30%	66%	71%	-	-	-	-	-	-
**Oryctolagus cuniculus - Rabbit**	**68%**	**99%**	**76%**	**99%**	**66%**	**79%**	**66%**	**52%**	**70%**	**89%**
**Rattus norvegicus - Brown rat**	**68%**	**99%**	**65%**	**98%**	**78%**	**84%**	**76%**	**34%**	**75%**	**49%**
**Mus musculus - Mouse**	**67%**	**98%**	**70%**	**99%**	**72%**	**76%**	**77%**	**40%**	**74%**	**46%**
Procavia capensis - Hyrax	67%	97%	-	-	-	-	-	-	75%	50%
Tupaia belangeri - Tree shrew	67%	26%	64%	99%	-	-	-	-	-	-
Macropus eugenii - Wallaby	66%	28%	-	-	-	-	-	-	-	-
Ochotona princeps - Pika	65%	99%	71%	99%	-	-	-	-	-	-
Cavia porcellus - Guinea pig	65%	98%	67%	99%	75%	82%	74%	42%	-	-
Monodelphis domestica - Opossum	65%	45%	65%	80%	-	-	-	-	-	-
Erinaceus europaeus - European hedgehog	63%	64%	62%	91%	72%	68%	-	-	-	-
Dasypus novemcinctus - Armadillo	62%	29%	71%	99%	-	-	-	-	-	-
Dipodomys ordii - Kangaroo rat	61%	34%	65%	99%	-	-	-	-	-	-
Loxodonta africana - Elephant	61%	29%	64%	98%	-	-	-	-	-	-
**Total**	**35**		**30**		**21**		**17**		**10**	

MI, Max Identity (i.e. the higher value among the identity values of the BLAST output); QC, Query Coverage (i.e. portion of the query sequence that was recognized by some subject sequence of that species); P, Primate. Alpha, [GenBank:NC_000014:106053245-106054732] (1488 bp); repeat, [GenBank:NC_000014:106048991-106049802] (812 bp); HS3, [GenBank:NC_000014:106048351-106048676] (326 bp); HS1,2, [GenBank:NC_000014:106041545-106042009] (465 bp); HS4, [GenBank:NC_000014:106032614-106032974] (361 bp).

### Dot plot analysis of the 3'RR

**Figure 4:**
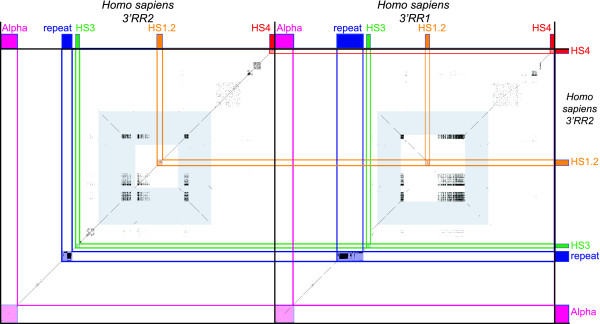
**Dot plot of the human 3'RR-2 against itself and 3'RR-1**. The two human 3'RR are compared to show the almost identical sequence and the palindromic structure encompassing the enhancer HS1.2 (light blue frame). Each portion of the palindrome is extended for ~3 kb. The black squares of spots indicate the presence of short stretches of DNA satellite tandem repeat. The two HS1.2 enhancers have inverted orientation in the two 3'RR as a result of the inverted orientation of the sequence internal to the palindrome. The orientation of the other enhancers is conserved and also the distances. The 20 bp repeat in dark blue appears to be larger in the 3'RR1.

**Figure 5:**
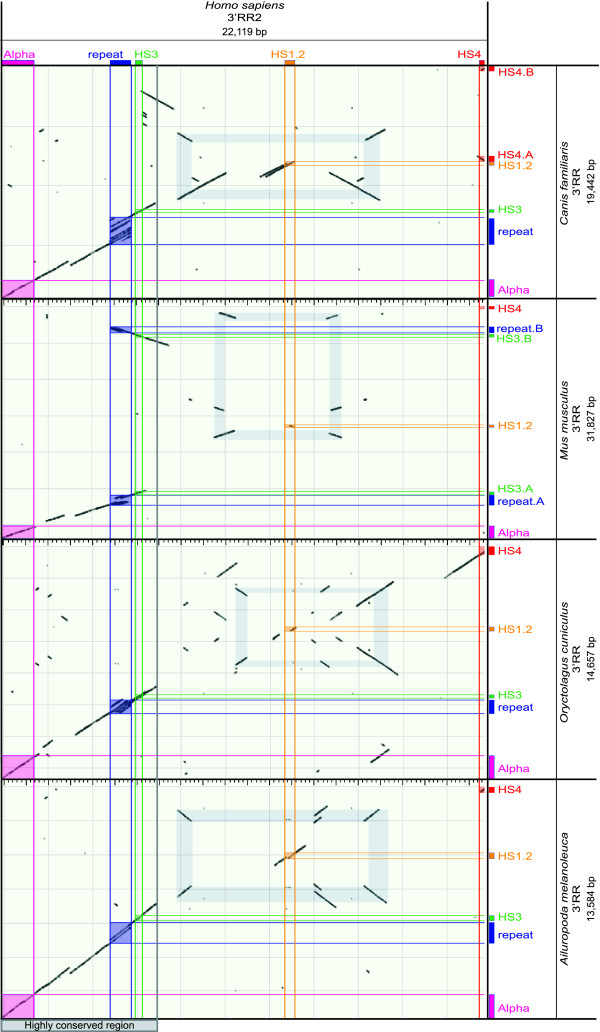
**Dot plot of the human 3'RR-2 against 4 species**. The comparison of the human regulatory region against the homologous sequences of *Ailuropoda melanoleuca (*panda), *Canis familiaris (*dog), *Mus musculus *(mouse) and *Oryctolagus cuniculus (*rabbit) shows the conservation of the enhancer positions and the high similarity in the sequence spanning from the alpha gene to the other extreme enhancer HS4. In the mouse the two copies of the enhancer HS3 A and B have in the neighbor region also the 20 bp repeat. In the mouse palindrome (light blue frame) are shown by the black segments with inverted orientation three copies of a region that is in two copies in humans. The wider extension of the palindrome in mouse includes the two HS3 enhancers on the boundaries. The same structures are conserved along the homologues 3'RR in these species and the palindrome is present in the cluster of all species as evidenced by the pale blue rectangle with the central region represented by the HS1.2 enhancer. The black lines depict the internal repeats present in more than one copy. The lines when parallel to the major diagonal have the same orientation, when are perpendicular have inverted orientation, as in the palindrome sequence.

It has been already reported, in man and mouse, that each HS1.2 enhancer is flanked, at some distance on both sides, by a 3 kb segment, and that these segments are in opposite orientation (palindromic), as evident from the dot plot analysis reported in Figure 4 (palindromic sequence in light blue). Very interestingly, we found that this organization is shared by 8 species in which the whole region was included in a fully assembled contig. The human versus non-primate dot-plots are reported in Figure 5. It is worth noting that, while the similarity between the two components of the same palindrome is always very high (94% in human, Figure 4), the sequence itself almost completely varied among species (Figure 5). Interestingly, HS1.2 always lies in the center of the palindrome. In the human 3' RR1, the region internal to the two components of the palindrome is inversely oriented with respect to the corresponding sequence of 3'RR2, as shown by the secondary diagonal line present at the core of the light blue frame (Figure 4). In addition, sequence comparisons of the human 3'RRs with non-primate mammals harboring a single copy of 3'RR (panda, rabbit, mouse, and dog) showed that in 3 of them, with the exception of the mouse, the orientation of the region internal to the palindrome (containing the HS1.2) was identical to the human 3'RR2 (Figure 5). This finding suggests that the 3'RR2 is ancestral with respect to the 3'RR1. The mouse showed an opposite orientation of the region internal to the palindrome. Moreover the mouse palindrome is larger than the human one, including the HS3 and the 20 bp repeat, thus originating HS3.A and HS3.B (Figure 5). Very likely, an inversion event was triggered by the palindrome both in the mouse 3'RR and in the human 3'RR1.

### HS1.2 enhancer in Trace Archive

While no polymorphisms have been reported for the enhancers HS3 and HS4[28], the 2 human HS1.2 copies share a set of variant forms (Figure 2)[21,29]. The main polymorphic feature of human HS1.2 consists of a tandem repeated pair of elements, i.e. a 40 bp sequence (40 mer, yellow boxes in Figure 2) and a ~15 bp cytosine-rich stretch (green boxes), that can or cannot be separated from the enhancer core (purple boxes) by a 29 bp sequence (red boxes). The HS1.2 human variants with more copies of the 40 mer showed an increasing effect on the transcription of a reporter gene in transfected cells[30]. In mouse there is just one copy of HS1.2 that constantly harbors a single copy of the 40 mer.

**Figure 6:**
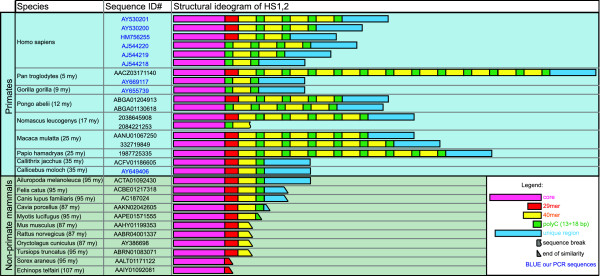
**Representation of the HS1.2 variants in 20 different species**. The alignment of the HS1.2 enhancer clones of the different species is shown by schematic representation. The conserved elements from the 5' towards the 3' are: the core constant part of the enhancer (fuchsia), the 29 mer sequence (red), the 40 mer (yellow), the stretch of cytosines (green), and the 3' end region (light blue) (see also Figure 2). The number of 40 mer copies appears variable in most of the primates. In these species the alleles with different number of the 40 mer can harbor either the 29 mer or the cytosine stretch between the core and the first 40 mer. Alleles with the 29 mer are present in the major part of the species. We found only clones with the cytosine stretch at the place of the 29 mer just in Gorilla. The identification number (ID) of each sequence is reported in the second column: the ID including a letter can be found in GenBank http://www.ncbi.nlm.nih.gov/Entrez/, the others in the Trace Archive database http://www.ncbi.nlm.nih.gov/Traces/trace.cgi. The bold characters are used to indicate sequences obtained in our laboratory.

The Trace Archive databases of primate species sequences was searched by BLAST using the human HS1.2 sequence as query, to investigate the evolutionary history of this enhancer and to search for potential polymorphisms. Figure 6 summarizes the obtained results, along with all the available data from previous works[21] and from our previously unpublished sequencing data. This figure shows the organization of the HS1.2 in the different species. The highly conserved core of the enhancer (113 bp, purple) is constantly flanked, in the 11 non-primate mammalian species (olive green background), by a partial 29 mer stretch (red element). The 102 bp terminal element (blue in Figure 6), constantly found in primates, was entirely detected only in panda when searched in non-primate mammals.

The most interesting finding of this analysis is the presence of a variable number of copies of the 40 mer (yellow in Figure 6) in all Catarrhini parvorder species, i.e. in both Hominoidea and Cercopithecoidea superfamilies. On the contrary, the duplication of the whole locus of the constant genes was found only in Hominoidea[3]. This observation strongly suggests that the emergence of the polymorphism occurred earlier with respect to the duplication. The number of the 40 mer varies from the 12 copies found in chimpanzee HS1.2, to a single one, as detected in some alleles in human, chimpanzee, and gorilla and in all the non-primate mammals. An additional variability found in Hominoidea is the occasional absence of the 29 mer, replaced by an 18 bp stretch of cytosine (green in Figure 6).

### HS1.2 Transcription Factor Binding Sites (TFBS)

All the HS1.2 forms found in the different species (Figure 6) were searched for transcription factor binding sites, using Alibaba2 software. Relevant results are summarized in Additional file 1 (full list in Additional file 2). Four TFBS (C/EBPalp, AP-2alpha, SP1, Oct1) are present in all the analyzed species; moreover, the NF variants are almost ubiquitous. The Additional file 1 clearly indicates that, while the number of C/EBPalp and Oct1 TFBS is substantially constant in different HS1.2 forms, the number of AP-2alpha and SP1 TFBS is proportional to the copies of the 40 mer present in that specific HS1.2 form. Note that c-myc containing 40 mers appears only in the Catarrhini HS1.2 forms and in dog.

### Phylogenetic analysis

**Figure 7:**
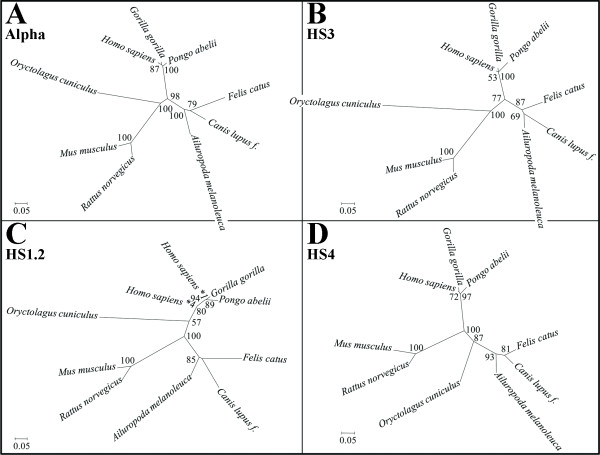
**Phylogenetic analysis of the C-alpha gene, HS3, HS1.2 and HS4 enhancers**. Unrooted phylogenetic trees for C-Alpha (A), HS3 (B), HS1.2 (C) and HS4 (D). Branch length is scaled according to the evolutionary distance, shown as the number of base substitutions per site. The percentage of 100 replicate trees in which the taxa clustered together after the bootstrap analysis is shown at the root of the branches when significant (i.e. when higher than 50%). As examples, the value '100' at the base of the separation between the taxa *Mus musculus *and *Rattus norvegicus *in panel D means that the two taxa were clustered together in all the replicate trees, while '72' between *Homo sapiens *and *Gorilla gorilla *in the same panel means that 72% of the 100 replicate trees clustered these two groups together.

The four structures clustered in the 3'RR were analyzed for their sequence variation in 9 species comprising human, gorilla, orangutan, mouse, rat, rabbit, panda, dog and cat (see Additional file 3). The phylogenetic analysis obtained with the maximum likelihood method for C-alpha, HS3, HS1.2 and HS4 (Figure 7A, B, C and 7D respectively), showed in all cases a similar variation from the standard reconstruction of mammals' phylogeny. Rodents and lagomorphs diverged from each other and from primates and carnivores, confirming at the nucleotide level the hypothesis of different evolutionary routes taken from the different groups, as shown after structural analysis. The concordance between the coding region (C-alpha) and the three enhancers in the observed divergence furthermore indicates that similar forces shaped the evolution of the whole 3' regulatory region, suggesting potential functional constrains also for the non coding sections.

## Discussion

In the present article we have compared the genomic structure of the 3'RR domain of the IgH gene cluster in various species. We have confirmed that in all the analyzed species the 3'RR elements order is largely maintained. Two main results were also achieved: (i) a palindromic structured sequence flanks each HS1.2 enhancer; (ii) HS1.2 is polymorphic in all analyzed Catarrhini species and therefore rose before of the IgH locus duplication.

### Palindromic sequences flanking the HS1.2

**Figure 8:**
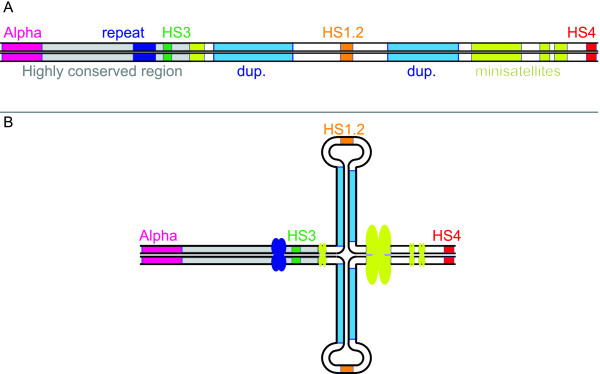
**Structural model of the 3'RR palindrome-driven internal hairpin**. This figure shows (A) the linear map of human 3'RR2 and (B) the hairpin loop shape that this sequence can putatively assumes. The whole HS1.2 enhancer (i.e. core, 29 mer, 40 mers and 3'end sequence) is here sketched as orange box. We did not show the HS1.2 orientation in this graphic because it differs between the two copies of the 3'RR in human, and among the 3'RR of the species in Figure 5. The hypothetical hairpin structure is independent from the orientation of HS1.2.

The most relevant result of our analysis was the finding that each HS1.2 enhancer, in all the examined species, is flanked by two 3 kb segments forming a palindromic structure (see Figures 4 and 5). Impressively, while the similarity of each pair of segments is extremely high, the similarity of the palindromic sequences among the different species is strikingly low. These findings suggest that the evolutionary pressure was much higher in maintaining the palindromic structure rather than the sequence conservation. As a consequence, it can be concluded that the palindrome plays a conformational role in the 3'RR functioning. The fact that the HS1.2 enhancer is constantly placed in the middle of the sequence spacing the two inverted elements, further supports the crucial role of the conformation of the region. We hypothesize that the palindrome triggers the formation of a hairpin structure externally exposing the HS1.2 enhancer (Figure 8). The orientation of HS1.2 is therefore irrelevant for the enhancer function. Indeed, it was found in different orientation in different species and also in different orientation in the two 3'RR human domains. Moreover, the opposite orientations of the two HS1.2 in human add support to the actual formation of the hairpin in vivo. The paired inverted sequences could form the stem of the hairpin. This is a fragile site that could be involved in rearrangements and translocation effect as in c-myc relocation[31]. An exchange involving the stem may result in the inversion of the loop region, changing the HS1.2 orientation. We can hypothesize that an inversion occurred at least two times since the divergence between *Homo sapiens *and *Mus musculus *(as suggested by dot plot analysis, see Figure 5 and above in Results). The inversions limits at least partially spanned the two palindromic regions, suggesting a cause/effect relationship. The palindrome could facilitate the inversion event, and the latter could contribute to perpetuate the palindrome.

### HS1.2 polymorphisms

Population genetics of HS1.2 polymorphisms is available only in humans, for which six distinct variants have been sequenced (AY530201, AY530200, AJ544220, AJ544219, AJ544218[21]; HM756255, our previously unpublished data). The human variants result from (i) a variable number of the 40 mer and its flanking cytosine-rich box (yellow and green, respectively, in Figure 6); (ii) the sequence connecting the constant core of the HS1.2 (purple in Figure 6) to the stretches of 40 mer repeats, that is constituted by a 18 bp cytosine-rich box or by a 29 mer (green and red, respectively, in Figure 6). HS1.2 polymorphisms have been detected also in 6 out of 8 non-human primates for whom this enhancer was identified in genomic databases (Figure 6). We acknowledge that the number of individuals for each species present in these archives is not known, as well as the sequence coverage. We can suppose that very few individuals, maybe a single one, are present in GenBank or Trace Archive. The variants we have detected are, therefore, very likely just the most frequent ones of each species. Nevertheless, it is worth noting that the two Platyrrhini (marmoset and titi) share the same HS1.2 form of the panda. We then hypothesize that this shared form of the enhancer was also at the base of all the Catarrhini variants.

IgA and 3'RR have relevance in response to infections and in diseases[3,16-18,20,32]. The more remarkable polymorphism found in 3'RR lies within the HS1.2 that has the central position in the palindromic structure, on top of the hairpin (Figure 8). We hypothesize that it can influence the modulation of the Ig switch through an interaction between the extruded enhancer and peptidic factors. The resulting molecular complexes may affect the mobility of the entire 3'RR and finally the formation of loops joining different constant and variable Ig portions. It could be interesting to investigate the role of the variants we have found in differently modulating the Ig switch and production in different species, especially in animal models such as macaque and mouse.

## Conclusions

We remark that both coding sequences and wide non-coding regulatory regions have undergone to some evolutionary pressure, and that part of this pressure was aimed to preserve the 3'RR three-dimensional structure for the conservation of the regulatory function necessary for class switch recombination[12].

## Methods

### Conserved synteny screening

We used the following list of mouse sequence tags from [GenBank:NC_000078.5]: a 20 bp tandem repeat (114493451-114493793), an alpha locus portion (114496952-114497890) and seven enhancers, that is HS1.2 (114483000-114483159), HS3.A (114470545-114470747), HS3.B (114492057-114492261), HS4 (114466607-114466729), and HS5, HS6 and HS7. Those last three were localized by their primers sequences, listed in previous paper[33].

These sequences were aligned by BLAT (BLAST-Like Alignment Tool, http://genome.ucsc.edu/cgi-bin/hgBlat) versus the 2007 release of the *Mus musculus (*mouse) genome, to identify the limits of the mouse 3'RR contig (chr12:114,459,657-114,497,890 in mmu9 draft; size 38234 bp)(Figure 3). This region was used to investigate the Amniota species of which the sequenced genome was available in the "Comparative Genomics" group of tracks at the UCSC mouse genome browser site http://genome.ucsc.edu/cgi-bin/hgGateway?db=mm9. So the list of the species we checked was in the order: rat (*Rattus norvegicus*, rn4), Guinea pig (*Cavia porcellus*, cavPor3), man (*Homo sapiens*, hg19), chimpanzee (*Pan troglodytes*, panTro2), orangutan (*Pongo abelii*, ponAbe2), macaque (*Macaca mulatta*, rheMac2), marmoset (*Callithrix jacchus*, calJac3), dog (*Canis familiaris*, canFam2), panda (*Ailuropoda melanoleuca*, ailMel1), horse (*Equus caballus*, equCab2), cow (*Bos taurus*, bosTau4), rabbit (*Oryctolagus cuniculus*, oryCun2), elephant (*Loxodonta africana*, loxAfr3), opossum (*Monodelphis domestica*, monDom5), platypus (*Ornithorhynchus anatinus*, ornAna1), lizard (*Anolis carolinensis*, anoCar1), chicken (*Gallus gallus*, galGal3). We reported in parenthesis the scientific name and the genome draft code.

Inspecting the homologous human region in the UCSC human genome browser http://genome.ucsc.edu/cgi-bin/hgTracks?db=hg18, we searched the tracks related to the Neanderthal genome [27] for the presence of HS3, HS1.2 and HS4.

### Database searches for enhancers

We searched for clones with the mouse (see previous section) and human version of the Alpha portion, repeat and enhancers (Alpha, GenBank:NC_000014:106053245-106054732, 1488 bp; repeat, GenBank:NC_000014:106048991-106049802, 812 bp; HS3, GenBank:NC_000014:106048351-106048676, 326 bp; HS1,2, GenBank:NC_000014:106041545-106042009, 465 bp; HS4, GenBank:NC_000014:106032614-106032974, 361 bp), using the nucleotide version of BLAST (Basic Local Alignment Search Tool, http://blast.ncbi.nlm.nih.gov/Blast.cgi). The asked databases were from the "Others" group (i.e. limiting results to non-human and non-mouse records), further specifying in the Taxonomy field to exclude "Homo (taxid:9605)". The three checked databases were: refseq_genomic (fully-sequenced genome entries from NCBI's Reference Sequence project); wgs (whole genome shotgun sequence); htgs (high throughput genomic sequences). To allow a better matching among so divergent species, especially among their 3'end tails, we chose a non-standard set of parameters: Match/Mismatch Scores 1/-1; Existence/Extension Gap Costs 0/2. Then we chose from the BLAST output the relevant sequences (see Table 1 for the list of species with sequences similar to the human queries) by empirically setting the "Expect" threshold-value to 1e-05.

### Dot plot analysis

We identified 2 genomic contigs of *Homo sapiens *(3'RR1, [GenBank:NC_000014.8:106152458-106175002], and 3'RR2, [GenBank:NC_000014.8:106032614-106054732]), and one contig of *Mus musculus *[GenBank:NC_000078.5:114466607-114497890], *Oryctolagus cuniculus *[GenBank:AY386698.1:7160-21816] and *Canis lupus familiaris *[GenBank:AC187024.23:146314-165755]. Finally, we assembled a contig of *Ailuropoda melanoleuca*, sewing the scaffold3005_4 [GenBank:ACTA01092430.1:1574-6589] and the scaffold3005_5 [GenBank:ACTA01100430.1:1-7968] by a stretch of 600 N. We performed a series of pairwise comparisons among these six genomic contigs, by use of two dot plot analysis softwares: Gepard http://mips.gsf.de/services/analysis/gepard[34] for the human versus human dot plots (Figure 4), and Blast2seq http://www.ncbi.nlm.nih.gov/blast/bl2seq/wblast2.cgi for the human versus others species dot plots (Figure 5). Graphics were edited using Adobe Illustrator.

### Database hunting for HS1.2 polymorphisms

The search for HS1.2 polymorphisms was refined using the specialized alignment tool Trace-Archive BLAST http://blast.ncbi.nlm.nih.gov/Blast.cgi?BLAST_SPEC=TraceArchive&BLAST_PROGRAMS=megaBlast&PAGE_TYPE=BlastSearch. The Trace Archive http://www.ncbi.nlm.nih.gov/Traces/trace.cgi is a repository of sequencing data from gel/capillary platforms, partitioned by genome and sequencing methods. There is a daily-growing amount of available reads from thousands of WGS projects. We performed a BLAST search on every available database referring to species identified in the previous analysis for the presence of this enhancer and to the 24 primates available in the Archive (*Aotus nancymaae, Ateles geoffroyi, Callicebus moloch, Callithrix jacchus, Cercopithecus aethiops, Colobus guereza, Eulemur macaco, Gorilla gorilla, Homo sapiens, Hylobates concolor, Lemur catta, Macaca fuscata, Macaca mulatta, Microcebus murinus, Nomascus leucogenys, Otolemur garnettii, Pan paniscus, Pan troglodytes, Papio anubis, Papio cynocephalus, Papio hamadryas, Pongo pygmaeus abelii, Saimiri boliviensis, Tarsius syrichta*). The sequences detected were selected for HS1.2 completeness and multi-aligned by use of ClustalW (http://www.ebi.ac.uk/Tools/clustalw2/index.html[35]) and MAFFT (http://align.bmr.kyushu-u.ac.jp/mafft/software/[36]) and by use of Seaview http://pbil.univ-lyon1.fr/software/seaview.html to manual revise the ClustalW/MAFFT outputs. See Figure 6 for a graphical representation of the detected HS1.2 variants.

### Transfac analysis for transcription factors

The search for the transcription factor consensus was performed on the variant sequences of HS1.2 by the software AliBaba2.1 http://www.gene-regulation.com/pub/programs/alibaba2/index.html. Additional file 1 lists the transcription factors detected at least in ten loci. The full list can be inspected as Additional file 2.

### Phylogenetic analysis

Sequences of IgH constant alpha genes and of the enhancers HS3, HS1.2, HS4 retrieved after BLAST search, were used for the phylogenetic analysis (Figure 7; accession numbers and limits reported in Additional file 3). Multiple alignments of the sequences were obtained with Opal[37] and the results were manually inspected. The best-fitting substitution model was selected using ModelGenerator [38], under the Akaike information criterion (AIC1), as implemented in MultiPhyl online[39]. The following models were integrated in the phylogenetic analysis: GTR + I + G for C-alpha; HKY + I for HS3 and HS4; HKY + G for HS1.2.

An unrooted tree was constructed using the maximum likelihood method applied to nucleotides, as implemented in Garli version 0.96 http://www.bio.utexas.edu/faculty/antisense/garli/Garli.html, with bootstrap percentages obtained as a consensus after 100 replicates.

## Authors' contributions

PD'A was the main researcher and performed comparative genomics analysis, database searches, dot plot analysis and data interpretation and participated in manuscript writing and editing. MS carried out phylogenetic analysis, data interpretation, and participated in manuscript editing. VG sequenced the DNA samples, investigated the presence of Transcription Factor Binding Site, and participated in manuscript editing. MR participated in planning the experiment, in data interpretation, and in manuscript writing and editing. DF ideated the work and planned the experiments, and participated in manuscript writing and editing. All authors read and approved the final manuscript.

## List of nonstandard abbreviations

IgH: Immunoglobulin heavy chain; 3'RR: 3' Regulatory Region; TFBS: Transcription Factor Binding Sites.

## Additional files

**Note: **in the three additional files, the Accessions including at least one letter are picked from GenBank http://www.ncbi.nlm.nih.gov/Entrez/, the others including just numbers from the Trace Archive database http://www.ncbi.nlm.nih.gov/Traces/trace.cgi.

## Supplementary Material

Additional file 1 **Identified Transcription Factor Binding Sites (listed if found more than 10 time)**. Click here for file

Additional file 2 **Full list of identified Transcription Factor Binding Sites**. Click here for file

Additional file 3 **List of sequences used for phylogenetics analysis in figure **8. Click here for file

## References

[B1] Stavnezer J Amemiya CT Evolution of isotype switching Semin Immunol 2004 16 4 257 275 10.1016/j.smim.2004.08.005 15522624

[B2] Cognè M Birshtein BK Honjo T, Alt FW, Neuberger MS Regulation of Class Switch Recombination Molecular biology of B cells 2004 New York: Elsevier 289 305

[B3] Woof JM Kerr MA IgA function--variations on a theme Immunology 2004 113 2 175 177 10.1111/j.1365-2567.2004.01958.x 15379977 PMC1782559

[B4] Pettersson S Cook GP Bruggemann M Williams GT Neuberger MS A second B cell-specific enhancer 3' of the immunoglobulin heavy-chain locus Nature 1990 344 6262 165 168 10.1038/344165a0 2106628

[B5] Lieberson R Giannini SL Birshtein BK Eckhardt LA An enhancer at the 3' end of the mouse immunoglobulin heavy chain locus Nucleic acids research 1991 19 4 933 937 10.1093/nar/19.4.933 1901991 PMC333734

[B6] Chauveau C Cogne M Palindromic structure of the IgH 3'locus control region Nature genetics 1996 14 1 15 16 10.1038/ng0996-15 8782813

[B7] Saleque S Singh M Little RD Giannini SL Michaelson JS Birshtein BK Dyad symmetry within the mouse 3' IgH regulatory region includes two virtually identical enhancers (C alpha3'E and hs3) Journal of immunology 1997 158 10 4780 4787 9144492

[B8] Chen C Birshtein BK Virtually identical enhancers containing a segment of homology to murine 3'IgH-E(hs1,2) lie downstream of human Ig C alpha 1 and C alpha 2 genes J Immunol 1997 159 3 1310 1318 9233627

[B9] Laurencikiene J Tamosiunas V Severinson E Regulation of epsilon germline transcription and switch region mutations by IgH locus 3' enhancers in transgenic mice Blood 2007 109 1 159 167 10.1182/blood-2006-02-005355 16968901

[B10] Gostissa M Yan CT Bianco JM Cogne M Pinaud E Alt FW Long-range oncogenic activation of Igh-c-myc translocations by the Igh 3' regulatory region Nature 2009 462 7274 803 807 10.1038/nature08633 20010689 PMC2802177

[B11] Vincent-Fabert C Fiancette R Cogne M Pinaud E Denizot Y The IgH 3' regulatory region and its implication in lymphomagenesis Eur J Immunol 40 12 3306 3311 10.1002/eji.201040778 21080376

[B12] Wuerffel R Wang L Grigera F Manis J Selsing E Perlot T Alt FW Cogne M Pinaud E Kenter AL S-S synapsis during class switch recombination is promoted by distantly located transcriptional elements and activation-induced deaminase Immunity 2007 27 5 711 722 10.1016/j.immuni.2007.09.007 17980632 PMC4979535

[B13] Dunnick WA Collins JT Shi J Westfield G Fontaine C Hakimpour P Papavasiliou FN Switch recombination and somatic hypermutation are controlled by the heavy chain 3' enhancer region J Exp Med 2009 206 12 2613 2623 10.1084/jem.20091280 19887393 PMC2806627

[B14] Vincent-Fabert C Fiancette R Pinaud E Truffinet V Cogne N Cogne M Denizot Y Genomic deletion of the whole IgH 3' regulatory region (hs3a, hs1,2, hs3b, and hs4) dramatically affects class switch recombination and Ig secretion to all isotypes Blood 2010 116 11 1895 1898 10.1182/blood-2010-01-264689 20538806

[B15] Giambra V Volpi S Emelyanov AV Pflugh D Bothwell AL Norio P Fan Y Ju Z Skoultchi AI Hardy RR Pax5 and linker histone H1 coordinate DNA methylation and histone modifications in the 3' regulatory region of the immunoglobulin heavy chain locus Mol Cell Biol 2008 28 19 6123 6133 10.1128/MCB.00233-08 18644860 PMC2547000

[B16] Frezza D Giambra V Mattioli C Piccoli K Massoud R Siracusano A Di Giannantonio M Birshtein BK Rubino IA Allelic frequencies of 3' Ig heavy chain locus enhancer HS1,2-A associated with Ig levels in patients with schizophrenia Int J Immunopathol Pharmacol 2009 22 1 115 123 19309558 10.1177/039463200902200113 PMC2721332

[B17] Giambra V Cianci R Lolli S Mattioli C Tampella G Cattalini M Kilic SS Pandolfi F Plebani A Frezza D Allele *1 of HS1.2 enhancer associates with selective IgA deficiency and IgM concentration J Immunol 2009 183 12 8280 8285 10.4049/jimmunol.0902426 20007591

[B18] Tolusso B Frezza D Mattioli C Fedele AL Bosello S Faustini F Peluso G Giambra V Pietrapertosa D Morelli A Allele *2 of the HS1,2A enhancer of the Ig regulatory region associates with rheumatoid arthritis Ann Rheum Dis 2009 68 3 416 419 10.1136/ard.2008.095414 18952640 PMC2633630

[B19] Cianci R Giambra V Mattioli C Esposito M Cammarota G Scibilia G Magazzu G Orlando A Sandri G Bianchi L Increased frequency of Ig heavy-chain HS1,2-A enhancer *2 allele in dermatitis herpetiformis, plaque psoriasis, and psoriatic arthritis J Invest Dermatol 2008 128 8 1920 1924 10.1038/jid.2008.40 18323783

[B20] Frezza D Giambra V Cianci R Fruscalzo A Giufre M Cammarota G Martinez-Labarga C Rickards O Scibilia G Sferlazzas C Increased frequency of the immunoglobulin enhancer HS1,2 allele 2 in coeliac disease Scand J Gastroenterol 2004 39 11 1083 1087 10.1080/00365520410007999 15545166

[B21] Giambra V Fruscalzo A Giufre M Martinez-Labarga C Favaro M Rocchi M Frezza D Evolution of human IgH3'EC duplicated structures: both enhancers HS1,2 are polymorphic with variation of transcription factor's consensus sites Gene 2005 346 105 114 10.1016/j.gene.2004.10.009 15716094

[B22] Chauveau C Cogne M Palindromic structure of the IgH 3'locus control region Nat Genet 1996 14 1 15 16 10.1038/ng0996-15 8782813

[B23] Saleque S Singh M Little RD Giannini SL Michaelson JS Birshtein BK Dyad symmetry within the mouse 3' IgH regulatory region includes two virtually identical enhancers (C alpha3'E and hs3) J Immunol 1997 158 10 4780 4787 9144492

[B24] Sepulveda MA Garrett FE Price-Whelan A Birshtein BK Comparative analysis of human and mouse 3' Igh regulatory regions identifies distinctive structural features Mol Immunol 2005 42 5 605 615 10.1016/j.molimm.2004.09.006 15607820

[B25] Meireles-Filho AC Stark A Comparative genomics of gene regulation-conservation and divergence of cis-regulatory information Curr Opin Genet Dev 2009 19 6 565 570 10.1016/j.gde.2009.10.006 19913403

[B26] Gambon-Deza F Sanchez-Espinel C Magadan-Mompo S The immunoglobulin heavy chain locus in the platypus (Ornithorhynchus anatinus) Mol Immunol 2009 46 13 2515 2523 10.1016/j.molimm.2009.05.025 19505725

[B27] Green RE Krause J Briggs AW Maricic T Stenzel U Kircher M Patterson N Li H Zhai W Fritz MH A draft sequence of the Neandertal genome Science 2010 328 5979 710 722 10.1126/science.1188021 20448178 PMC5100745

[B28] Guglielmi L Truffinet V Magnoux E Cogne M Denizot Y The polymorphism of the locus control region lying downstream the human IgH locus is restricted to hs1,2 but not to hs3 and hs4 enhancers Immunol Lett 2004 94 1-2 77 81 10.1016/j.imlet.2004.04.003 15234538

[B29] Mills FC Harindranath N Mitchell M Max EE Enhancer complexes located downstream of both human immunoglobulin Calpha genes J Exp Med 1997 186 6 845 858 10.1084/jem.186.6.845 9294139 PMC2199054

[B30] Denizot Y Pinaud E Aupetit C Le Morvan C Magnoux E Aldigier JC Cogne M Polymorphism of the human alpha1 immunoglobulin gene 3' enhancer hs1,2 and its relation to gene expression Immunology 2001 103 1 35 40 10.1046/j.1365-2567.2001.01217.x 11380690 PMC1783220

[B31] Osborne CS Chakalova L Mitchell JA Horton A Wood AL Bolland DJ Corcoran AE Fraser P Myc dynamically and preferentially relocates to a transcription factory occupied by Igh PLoS Biol 2007 5 8 e192 10.1371/journal.pbio.0050192 17622196 PMC1945077

[B32] Aupetit C Drouet M Pinaud E Denizot Y Aldigier JC Bridoux F Cogne M Alleles of the alpha1 immunoglobulin gene 3' enhancer control evolution of IgA nephropathy toward renal failure Kidney Int 2000 58 3 966 971 10.1046/j.1523-1755.2000.00253.x 10972660

[B33] Garrett FE Emelyanov AV Sepulveda MA Flanagan P Volpi S Li F Loukinov D Eckhardt LA Lobanenkov VV Birshtein BK Chromatin architecture near a potential 3' end of the igh locus involves modular regulation of histone modifications during B-Cell development and in vivo occupancy at CTCF sites Mol Cell Biol 2005 25 4 1511 1525 10.1128/MCB.25.4.1511-1525.2005 15684400 PMC548023

[B34] Krumsiek J Arnold R Rattei T Gepard: a rapid and sensitive tool for creating dotplots on genome scale Bioinformatics 2007 23 8 1026 1028 10.1093/bioinformatics/btm039 17309896

[B35] Thompson JD Gibson TJ Higgins DG Multiple sequence alignment using ClustalW and ClustalX Curr Protoc Bioinformatics 2002 **Chapter 2**: Unit 2 3 18792934 10.1002/0471250953.bi0203s00

[B36] Katoh K Asimenos G Toh H Multiple alignment of DNA sequences with MAFFT Methods Mol Biol 2009 537 39 64 full_text 19378139 10.1007/978-1-59745-251-9_3

[B37] Wheeler TJ Kececioglu JD Multiple alignment by aligning alignments Bioinformatics 2007 23 13 i559 568 10.1093/bioinformatics/btm226 17646343

[B38] Keane TM Creevey CJ Pentony MM Naughton TJ McLnerney JO Assessment of methods for amino acid matrix selection and their use on empirical data shows that ad hoc assumptions for choice of matrix are not justified BMC Evol Biol 2006 6 29 10.1186/1471-2148-6-29 16563161 PMC1435933

[B39] Keane TM Naughton TJ McInerney JO MultiPhyl: a high-throughput phylogenomics webserver using distributed computing Nucleic Acids Res 2007 35 Web Server W33 37 10.1093/nar/gkm359 17553837 PMC1933173

